# Seeing Is Believing: Real-Life 360-Degree Virtual Reality as a Catalyst for Neurosurgical Interest

**DOI:** 10.7759/cureus.82720

**Published:** 2025-04-21

**Authors:** Tatsuya Tanaka, Eiichi Suehiro, Shoichi Inagawa, Akira Matsuno

**Affiliations:** 1 Department of Neurosurgery, International University of Health and Welfare Narita Hospital, Narita, JPN; 2 Department of Nursing, International University of Health and Welfare Narita Hospital, Narita, JPN

**Keywords:** 360-degree video, career motivation, clinical training, educational technology, immersive learning, medical education, neurosurgery, simulation-based education, undergraduate medical students, virtual reality

## Abstract

Background: Traditional surgical education faces challenges such as limited visibility, physical space constraints, and restricted access for students. Virtual reality (VR) technology offers a promising alternative, especially with real-life 360-degree video, which can recreate immersive clinical experiences. This study investigates whether real-life 360-degree VR can enhance medical students' interest in neurosurgery and serve as an effective educational tool.

Methods: A VR-based educational program was developed using 360-degree video footage captured with Insta360 Pro cameras installed in the emergency department, operating room, and angiography suite. The content was incorporated into a neurosurgical clinical training course for 85 fifth-year medical students at the International University of Health and Welfare Narita Hospital in Narita, Japan. Students viewed the VR content through synchronized head-mounted displays. After the session, an anonymous questionnaire assessed usability, immersion, comprehension, enjoyment, and interest in neurosurgery. A paired samples t-test was used to compare students' interest in neurosurgery before and after the session.

Results: High levels of satisfaction were reported with regard to usability (95%) and immersion (97.6%). Most students agreed that the VR session improved their understanding (92.9%) and was enjoyable (95%). Interest in neurosurgery increased significantly following the session, with affirmative responses rising from 22 (26%) to 52 (61%) students (t=6.77; df=84; p<0.0001).

Conclusion: Real-life 360-degree VR is an effective supplementary tool for neurosurgical education. It offers immersive experiences that can enhance student engagement, understanding, and interest in neurosurgery. Broader implementation of this technology may address declining interest in the specialty and enrich clinical education.

## Introduction

Traditional clinical education has largely depended on in-person experiences. However, the outbreak of the COVID-19 pandemic has led to restrictions on face-to-face training, bringing virtual reality (VR) technology into focus as an alternative [1,2]. VR technology is evaluated as a tool that helps enhance the understanding of complex procedures and anatomical structures while providing experiences close to those in real clinical settings [3-11].

There are two primary technological approaches to VR-based education. The first approach uses computer graphics (CG) to scan human body data and render it in 3D, which is then placed in a VR environment [3,4,7]. Users interact with the objects through devices such as controllers. The main focus of this approach is training on skills and techniques, such as surgical planning and skill enhancement. It is most effective in scenarios aimed at improving individual skills. The second approach uses real-life footage in VR, where clinical settings are recorded with a 360-degree camera and converted into VR through dedicated applications [7-11]. In this format, the user is placed at the center of the VR environment and can freely look around in all directions. The key feature of this approach is that, since it is based on real footage, the clinical cases occurring in the setting are recorded as seen by the participants, allowing users to repeatedly experience them without being restricted by time or location.

Neurosurgery is a highly specialized field, and factors such as work-life balance and physician burnout create significant barriers, making it an unappealing field for medical students [12,13]. Work-life balance is a major concern for medical students, who tend to prefer specialties that offer a better lifestyle, such as radiology, dermatology, and ophthalmology [14,15]. As a result, fewer students aspire to become neurosurgeons [14,15]. Therefore, there is a need for educational programs that can increase students' interest in neurosurgery [16-18]. 

This study aims to develop a VR educational tool using real-life 360-degree VR technology, which allows students to virtually experience a clinical environment close to reality, and to evaluate the effectiveness of this VR tool for neurosurgery education. Specifically, this study seeks to clarify how clinical experiences through the VR tool impact medical students' understanding of and interest in neurosurgery.

## Materials and methods

Creation of the VR educational tool 

To develop the VR educational tool, we first established a shooting environment. Three hundred and sixty-degree cameras were installed in the emergency room, operating room, and angiography suite to record emergency procedures and clinical activities performed by experienced neurosurgeons on patients requiring urgent neurosurgical intervention.

**Figure 1:**
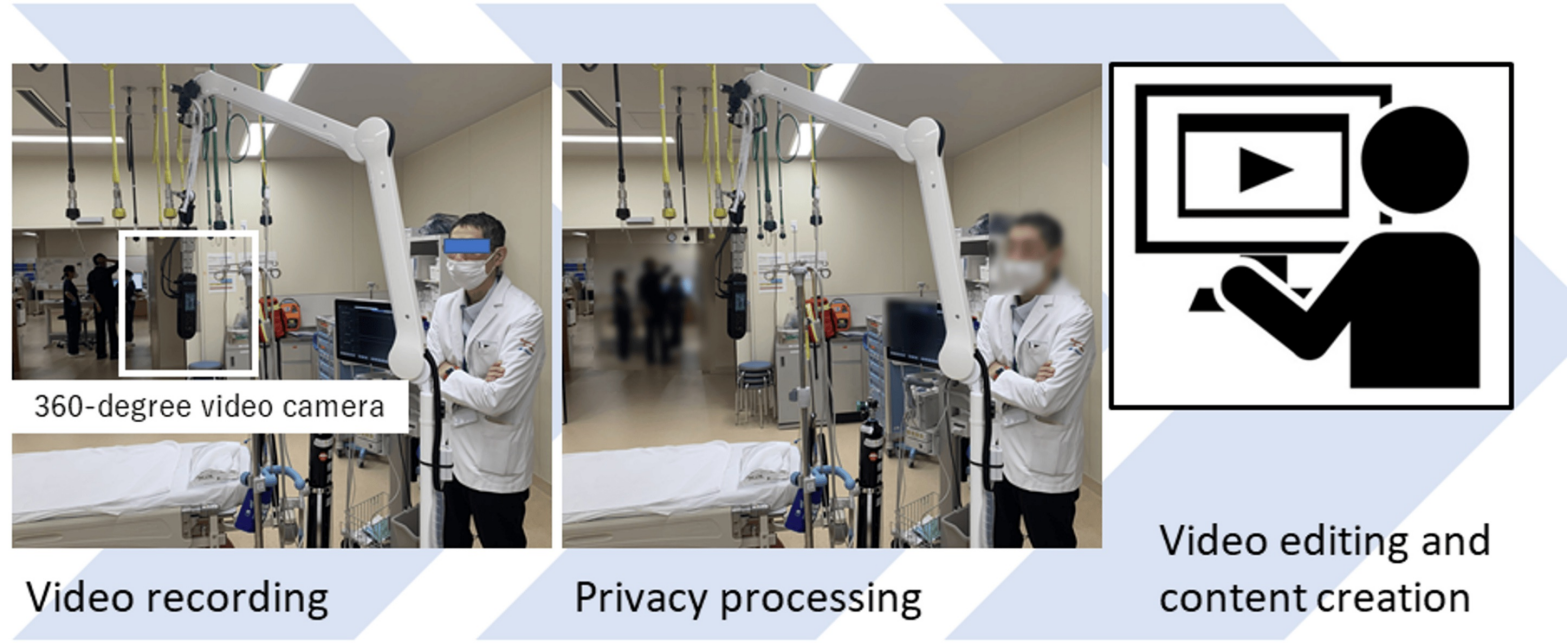
Workflow for creating educational 360-degree VR content. Step 1: Recording clinical scenes using a 360-degree video camera. Step 2: Privacy protection with masking of identifiable information. Step 3: Video editing and content creation. VR: virtual reality

The recorded footage underwent image processing that included mosaic effects and other privacy-preserving modifications. All recordings were made with the consent of the patients or their families. Edited using video editing software, the final product was designed to maximize immersion and realism, resulting in clinical VR content tailored for educational purposes (Figure 1).

**Figure 2:**
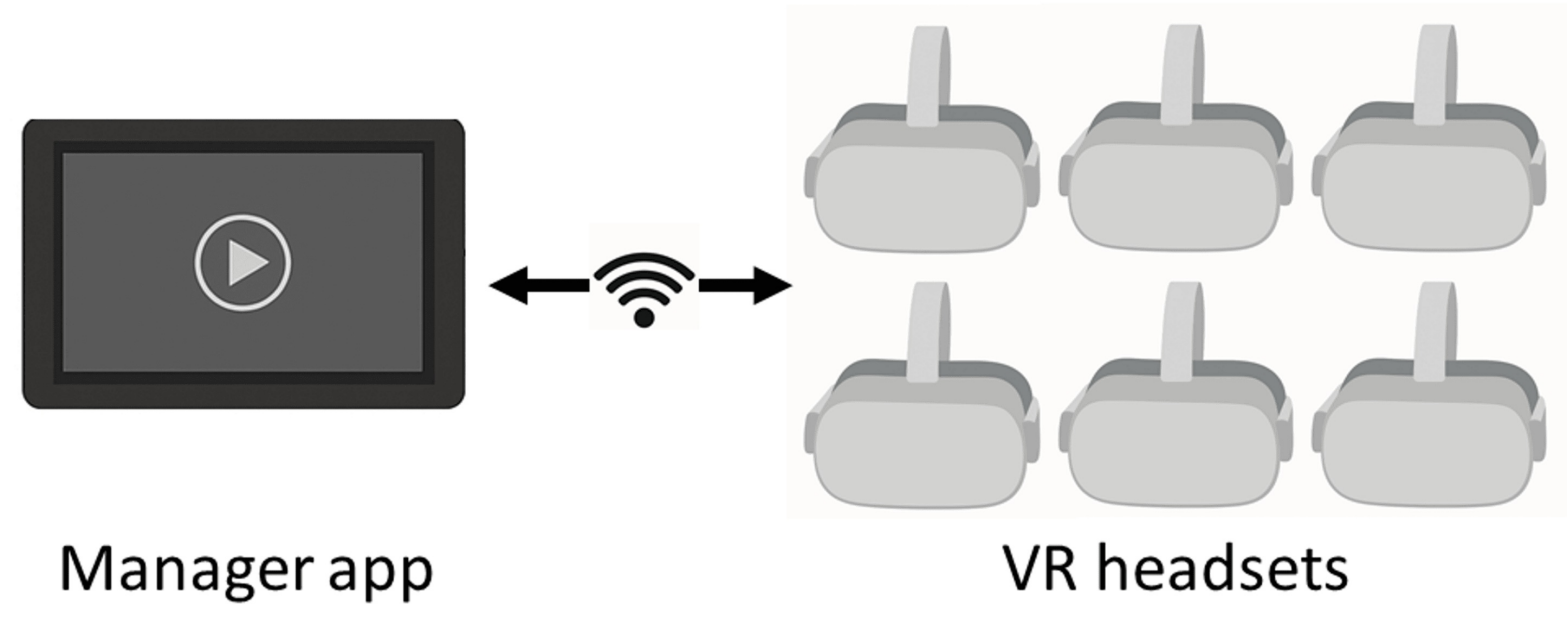
Synchronized control of multiple VR headsets. A manager app wirelessly controls the playback of VR content across multiple headsets. VR: virtual reality

To ensure a stable internet environment for viewing, we utilized a dedicated streaming server, routers, and LAN cables. The content was deployed in actual medical student education. The system, developed by Jolly Good Co., Ltd., incorporated the multi-mode function of GuruVR Smart Sync, enabling simultaneous viewing among multiple students and allowing real-time feedback and interaction (Figure 2).

VR-based classes and evaluation methods

Eighty-five fifth-year medical students (from April 2024 to March 2025) underwent a two-week clinical training program in the Department of Neurosurgery, International University of Health and Welfare Narita Hospital, Narita, Japan. Students were selected from among those assigned to the Department of Neurosurgery during their standard clinical rotation at Narita Hospital. Participation in the VR component was non-selective, and students received the same training experience as part of their curriculum.

**Figure 3:**
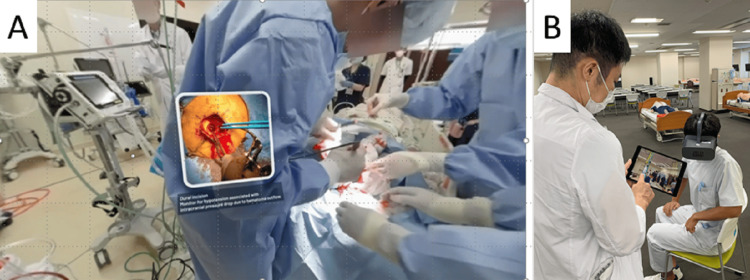
VR-based clinical education in small groups. (A) Students view immersive 360-degree surgical footage with educational overlays. (B) An instructor monitors a student's VR experience in real time using a synced tablet. VR: virtual reality

During the course, students attended classroom sessions conducted in small groups of six to seven members. Using VR goggles, students were exposed to real clinical scenarios, which fostered a sense of presence and urgency. GuruVR Smart Sync's multi-mode feature enabled instructors to monitor individual student viewpoints and provide timely guidance during the sessions (Figure 3).

To evaluate the educational impact, an anonymous questionnaire was administered after the VR class. The survey items assessed several aspects, including the ease of using the VR equipment, the immersive quality of the 360-degree real-life footage, the depth of understanding of the lecture content, the overall enjoyment of the session, and the students' interest in future VR-based classes. Responses were rated using a 5-point Likert scale. Furthermore, changes in students' interest in neurosurgery and their aspirations to specialize in the field before and after the session were measured.

For statistical analysis, a paired samples t-test was conducted to evaluate differences in students' interest in neurosurgery before and after the session, as the same cohort was assessed at two time points. A p-value of <0.05 was considered statistically significant.

Ethical considerations

This study was approved by the International University of Health and Welfare Ethics Committee (approval number: 23-Nr-017-1). The implementation of this VR-based tool did not alter the routine clinical procedures or the nature of the training provided. As such, the clinical content used for educational purposes was identical to standard care, and no additional physical or psychological burden was imposed on the participants.

## Results

Evaluation of the VR lecture

The ease of use of the VR equipment was highly rated by the participants. Specifically, 45 students (53%) strongly agreed and 36 students (42%) agreed that the VR equipment was easy to use, with only two students (2%) remaining neutral and two students (2%) disagreeing. Overall, approximately 95% of the students provided positive feedback regarding the usability of the VR equipment.

Regarding the sense of presence in the 360-degree real-life VR, 63 students (74%) strongly agreed and 20 students (24%) agreed that the VR experience provided a high level of immersion. Only one student (1%) remained neutral, and one student (1%) disagreed. In total, approximately 97.6% of the participants rated the sense of presence in the VR experience highly.

As for the understanding of the lecture content, 45 students (53%) strongly agreed and 34 students (40%) agreed that the VR experience enhanced their understanding of the lecture. Only four students (5%) remained neutral, and two students (2%) disagreed. In total, approximately 92.9% of the participants affirmed that their understanding of the lecture content was improved.

**Figure 4:**
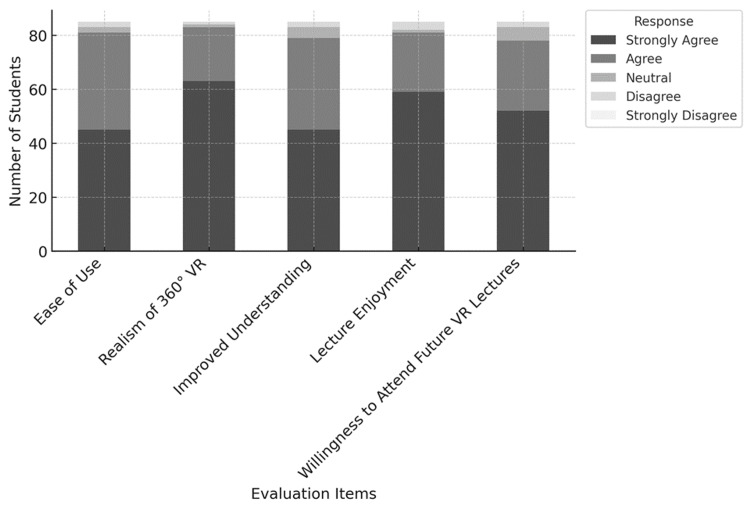
Student evaluation of a VR-based neurosurgery lecture. This figure shows student responses to a VR-based neurosurgery lecture. Most students agreed on the ease of use, immersive realism of 360-degree VR, improved understanding, lecture enjoyment, and willingness to attend future VR sessions. VR: virtual reality

In terms of the enjoyment of the lecture and the willingness to participate in future VR lectures, 59 students (69%) strongly agreed and 22 students (26%) agreed that they enjoyed the lecture. Only three students (4%) disagreed, and one student (1%) remained neutral. When asked whether they would like to participate in future VR lectures, 52 students (61%) strongly agreed and 26 students (30%) agreed, indicating that approximately 91.8% of the students were interested in continuing to participate in VR lectures (Table 1 and Figure 4).

**Table 1:** Student evaluation of a VR-based neurosurgery lecture. VR: virtual reality

Question	Strongly agree	Agree	Neutral	Disagree	Strongly disagree
The VR device was easy to use	45	36	2	2	0
The 360-degree real-life VR provided a sense of presence	63	20	1	1	0
VR improved my understanding of the lecture	45	34	4	2	0
The VR-based lecture was enjoyable	59	22	1	3	0
I would like to attend future VR-based lectures	52	26	5	2	0

Changes in awareness of neurosurgery and paired samples t‑test

Before the lecture, when asked if they considered neurosurgery as a specialty, 22 students (26%) responded positively ("Strongly agree" + "Agree"), while 63 students (74%) responded negatively or neutrally ("Neutral" + "Disagree" + "Strongly disagree").

After the lecture, the item "Interest in neurosurgery increased after participating in the VR lecture" showed a marked shift in responses. Fifty-two students (61%) reported a positive change in their interest in neurosurgery, while 33 students (39%) did not report any change or showed a decrease in interest.

**Figure 5:**
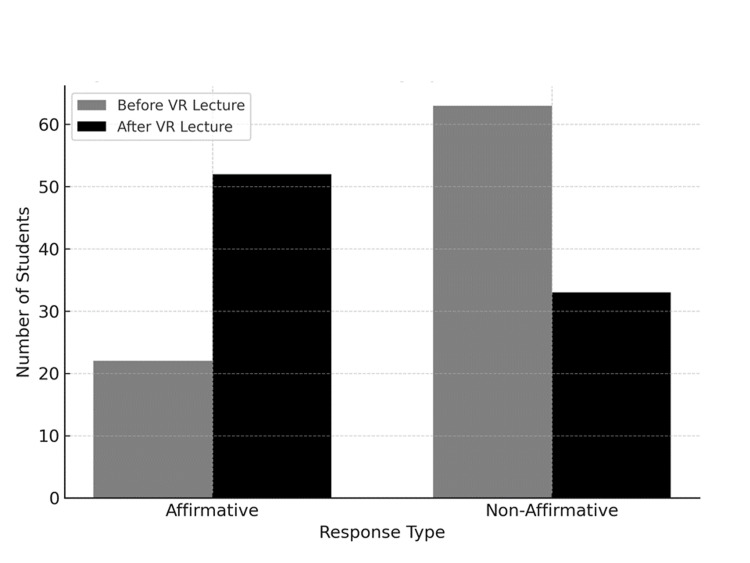
Change in interest in neurosurgery before and after the VR lecture. This figure shows a significant increase in students' interest in neurosurgery following the VR session (t=6.77; df=84; p<0.0001). Affirmative responses increased from 22 (26%) before the lecture to 52 (61%) after the lecture. VR: virtual reality

A paired samples t-test was conducted to examine the change in students' interest in neurosurgery before and after the VR session. The results showed a statistically significant increase in interest (t=6.77; df=84; p<0.0001), indicating that the VR lecture significantly increased students' interest in neurosurgery (Table 2 and Figure 5).

**Table 2:** Change in interest in neurosurgery before and after the VR lecture. VR: virtual reality

Question	Strongly agree	Agree	Neutral	Disagree	Strongly disagree
Before the VR lecture, I considered neurosurgery as a future specialty	12	10	22	33	8
My interest in neurosurgery increased after attending the VR lecture	19	33	26	4	3
I feel the VR lecture increased the likelihood of students pursuing neurosurgery	24	43	15	2	1

## Discussion

This study demonstrated that neurosurgical clinical training using real-life 360-degree VR technology provided medical students with high usability, immersion, enhanced understanding, and enjoyment, receiving very positive feedback overall. Notably, the significant increase in students' interest in neurosurgery before and after the VR training confirms that VR-based learning is an effective method for stimulating students' interest.

Traditionally, clinical education has relied heavily on in-person experiences, with direct exposure to clinical environments and complex procedures being considered essential for learning. However, due to the COVID-19 pandemic, restrictions on face-to-face training have led to an increased focus on VR technology as an alternative educational tool [1,2]. VR and simulation technologies have been shown to provide near-real clinical experiences that aid in knowledge retention and skill acquisition [1-11]. In particular, VR training has been beneficial in improving the learning motivation and practical abilities of students in fields such as medical education and clinical clerkships [1-11].

Moreover, VR technology holds significant potential to enhance the quality of clinical training environments and improve educational outcomes. Future advancements should address challenges such as cost, video editing techniques, and faculty training in the operation of VR systems. Additionally, cross-comparative studies of successful implementations across various fields are essential for further development.

The number of medical students aspiring to become neurosurgeons is decreasing globally, primarily due to poor work-life balance in the field [14,15]. The increasing stress and burnout among neurosurgeons further exacerbate this issue [12,13]. To address this, it is crucial to effectively communicate the appeal of neurosurgery to students and actively promote the specialty. Previous studies have shown that early exposure to neurosurgery can significantly increase student interest [16,18]. This study suggests that providing opportunities for students, even those initially uninterested in neurosurgery, to actively engage in the field is valuable, and non-selective participation in clinical training may be an effective strategy.

Considering the effectiveness of early exposure, incorporating neurosurgical training into the early stages of medical degree programs may increase the number of students pursuing neurosurgery as a specialty. Early intervention could lead to a more substantial number of aspiring neurosurgeons and help address the ongoing decline in interest in this field.

Limitations of the study

This study has several limitations. The most significant limitation is the lack of long-term follow-up. It remains unclear whether the increased interest in neurosurgery observed after the training is sustained over time or whether it leads to an increase in residents aspiring to pursue neurosurgery. Additionally, since the pre-training interest in neurosurgery was assessed through self-reporting after the training, recall bias should be considered. The inclusion of a control group could have provided a clearer evaluation of the effectiveness of the training. Furthermore, as this study was conducted at a single facility in Japan, the results may not be directly generalizable to other countries or regions. The environments surrounding medical students and neurosurgeons vary globally, so tailored approaches should be developed to address regional needs.

## Conclusions

This study demonstrated that neurosurgical clinical training using real-life 360-degree VR technology provided a level of immersion that was not achievable with traditional 2D materials or observational training. It contributed to enhancing students' understanding of and interest in clinical practice. Notably, the significant increase in positive perceptions of neurosurgery before and after the VR training indicates that VR-based learning is effective in motivating students to pursue specialization in neurosurgery. Additionally, consistent with findings from other fields where VR has been implemented, this study suggests that 360-degree VR technology can contribute to the overall improvement of the quality of medical education.
